# Supplementary material for: Physics of swimming and its fitness cost determine strategies of bacterial investment in flagellar motility
Source: Nat Commun. 2025 Feb 18;16:1731. doi: 10.1038/s41467-025-56980-x (PMC11836070; doi:10.1038/s41467-025-56980-x)
Supplement: Supplementary file 1 — Supplementary Information [file 41467_2025_56980_MOESM1_ESM.pdf]

Supplementary Information

Physics of swimming and its fitness cost determine strategies of bacterial investment in flagellar motility

Irina Lisevich[1], Remy Colin[1], Hao Yuan Yang[1,2], Bin Ni[1,3] & Victor Sourjik[1]

[1]Max Planck Institute for Terrestrial Microbiology & Center for Synthetic Microbiology (SYNMIKRO), Karl-von-Frisch-Strasse 14, Marburg, D-35043, Germany

[2]Max Planck School Matter to Life, Jahnstraße 29, Heidelberg, D-69120, Germany

[3]College of Resources and Environmental Science, National Academy of Agriculture Green Development, China Agricultural University, Yuanmingyuan Xilu No. 2, Beijing 100193, China

Supplementary Note 1

Differential Dynamic Microscopy

The principles of differential dynamic microscopy (DDM) and its application to measuring bacterial swimming behavior have been already described in detail[1-4]. Thus, we only briefly summarize the main points here.

The method exploits the rate of changes in pixel intensity in an image being proportional to the speed of motion of the object to measure it. The analysis is carried out in spatial Fourier space because the structure of the information is more easily exploitable in this format. We can write the pixel intensity of a movie frame featuring N moving bacteria in a static background I_{bg} as:

$$I(\mathbf{x},t) = I_{bg}(\mathbf{x}) + \sum_{i=0}^{N} I_p\big(z_i(t), \mathbf{x} - \mathbf{x}_i(t)\big) + \epsilon(\mathbf{x},t) \tag{1.1}$$

Where $\mathbf{x}_i(t)$ is the 2D position and $z_i(t)$ the position relative to the focal plane of bacterium i, $\epsilon(\mathbf{x},t)$ is the camera shot noise, and I_p is the pixelated image of the bacterium, that depends on the optics of the microscope and the camera and is centered around the position of the bacterium $\mathbf{x}_i(t)$. DDM first computes the spatial Fourier transform of this intensity:

$$I(\mathbf{q},t) = \int d\mathbf{x}\, e^{-i\mathbf{q}\cdot\mathbf{x}}\, I(\mathbf{x},t) \;= I_{bg}(\mathbf{q}) + \epsilon(\mathbf{q},t) + \sum_{i=0}^{N} e^{-i\mathbf{q}\cdot\mathbf{x}_i(t)}\, I_p(z_i(t), \mathbf{q}) \tag{1.2}$$

It then focusses on the differential intensity correlation function (DCIF):

$$D(\mathbf{q}, dt) = \langle |I(\mathbf{q}, t + dt) - I(\mathbf{q}, t)|^2 \rangle \tag{1.3}$$

Under the assumption that $I_p(z_i(t), \mathbf{q})$ evolves much more slowly than $e^{-i\mathbf{q}\cdot\mathbf{x}_i(t)}$, which has been verified for the low magnification optics we use and in the range of wave numbers $q = |\mathbf{q}|$ we consider[2,3], and in the case of a dilute system where the motion of different bacteria can be seen as independent from each other, we can write the DCIF as[1,2]:

$$D(\mathbf{q}, dt) = 2N\left|\bar{I}_p(\mathbf{q})\right|^2 S(\mathbf{q})\big(1 - f(\mathbf{q}, dt)\big) + \langle |\epsilon(\mathbf{q})|^2 \rangle \tag{1.4}$$

With the structure factor $S(\mathbf{q}) = \langle e^{-i\mathbf{q}\cdot(\mathbf{x}_i - \mathbf{x}_j)} \rangle_{i,j}$ that quantifies the spatial structure of the cell suspension, $\bar{I}_p(\mathbf{q})$ averaging over visible cells at different z, and the intermediate scattering function (ISF):

$$f(\mathbf{q}, dt) = \langle e^{-i\mathbf{q}\cdot\Delta\mathbf{x}(dt)} \rangle = \int d\mathbf{x}\, e^{-i\mathbf{q}\cdot\mathbf{x}}\, p(\mathbf{x}|dt) \tag{1.5}$$

Which is the Fourier transform of the probability of displacements $p(\mathbf{x}|dt)$ of the bacteria during a time step dt, and thus fully characterizes the dynamics of the system. The functional form of $f(\mathbf{q}, dt)$ is known for various types of motion, including swimming cells and diffusing non-motile cells.

Since our sample contains both motile and non-motile cells, we fit the experimentally determined DCIF with the following model[2]:

$$D(\mathbf{q}, dt) = A(q)\,\Big(1 - f_{ps}(\mathbf{q}, dt)\Big) + B(q) \tag{1.6}$$

With the intermediate scattering function accounting for only a fraction ϕ_M of cells being motile:

$$f_{ps}(\mathbf{q},dt) = \left(\phi_M f_M(\mathbf{q},dt) + (1-\phi_M)\right) f_B(\mathbf{q},dt) \tag{1.7}$$

The Brownian ISF accounts for the Brownian motion of the cells (primarily the non-motile ones):

$$f_B(\mathbf{q},dt) = \exp(-D_0 q^2 dt) \tag{1.8}$$

With D_0 their diffusion coefficient. This function decays from 1 to 0 in a typical time $dt \sim 1/D_0 q^2$.

The ISF of the motile cells has the form:

$$f_M(\mathbf{q},dt) = g(qv_0 dt \mid \sigma_v/v_0) \tag{1.9}$$

With v_0 the average swimming speed of the population and σ_v its variance. In the limit $\sigma_v = 0$, the function g is a cardinal sine, $g(x|0) = \sin x/x$. In Wilson *et al* (2011)[2], it was computed analytically in the case of Schultz distributed velocities, a model that proved proficient:

$$g(x \mid y) = \left(\frac{Z+1}{Zx}\right) \frac{\sin\left(Z \tan^{-1}\left(\frac{x}{Z+1}\right)\right)}{\left(1+\left(\frac{x}{Z+1}\right)^2\right)^{\frac{Z}{2}}} \tag{1.10}$$

With $Z = (y^2-1)/y^2$. In all cases, the function decays from 1 to 0 on a typical time $dt \sim 1/qv_0$.

In practice, the DCIF $D(q,dt)$, which is computed over the accessible range of q given our pixel size and the width of our camera field of view, displays two distinct increases as a function of dt (Supplementary Fig. 2a). These are respectively due to the motile cells (corresponding to the $1/qv_0$ decay timescale of f_{ps}) and the non-motile ones ($1/D_0 q^2$ timescale). The relative amplitudes of the two increases indicate the fraction of cells that are swimming (Supplementary Fig. 2a). Since the time scales have different dependences in q, we independently fit $D(q,dt)$ as a function of dt by the model (Eqs. 1.6-1.10) for different q, and verify the consistency of the fit parameters as a function of q (Supplementary Fig. 2b). We thus are able to validate the model and in particular the motile and Brownian nature of the motion of the two subpopulations. For very small and very large q, the fits fail, because the correlation function does not fully decorrelate over the duration of the experiment (small q) or because the signal over noise ratio is too small (large q). We thus reject the resulting very noisy values for the fitted parameters. We then use the average value of the parameters over the valid q range as the measured values for the given experiment, while the standard deviation measures the accuracy of our estimation of said parameters.

Dark Field Flicker Microscopy

Dark field flicker microscopy (DFFM) is a recently developed optical microscopy method, which exploits the specificity of dark field illumination to measure the rotation speed of the cell body and the flagellum[5,6]. Under dark field illumination, the objective collects only the light that is scattered by the microscopic objects that are in focus. In the regime of Mie scattering in which bacteria lie, the directions in which an *anisotropic* object (like the cell body or the flagellum) scatters incident light depends on the angle between the object main axis and the incident light. The rotation of this axis thus modifies the main scattering directions and therefore the amount of light collected by the objective: the image of the object flickers at

a frequency that is equal to the rotation frequency of the object. This flickering can thus be exploited to measure said frequency.

In practice, a bacterial suspension prepared as for DDM (OD_{600} ~ 0.1-0.2) is observed at 10x magnification (NA=0.3) under dark field illumination, and a 512 x 512 px^2 (1 px = 0.7 µm) field of view is recorded at 800 frames/s during 10^4 frames with an EoSens 4CPX CMOS camera, far from the sample surfaces. The movie is divided in independent 8x8 px^2 submovies, in which there is on average no more than one cell at a time. The temporal power spectrum of the average intensity of the submovies is computed as

$$S(\omega) = \langle |\overline{I_k}(\omega)|^2 \rangle_k \tag{1.11}$$

With $\overline{I_k}(\omega)$ the Fourier transform of the average intensity of submovie k:

$$\overline{I_k}(\omega) = \int dt\, e^{-i\omega t} \langle I(\mathbf{x}, t) \rangle_{\mathbf{x} \in V(k)} \tag{1.12}$$

The power spectrum is then corrected for the effects of the cells Brownian motion by computing:

$$E(\omega) = \omega^2 S(\omega) \tag{1.13}$$

For swimming *E. coli*, the corrected power spectrum $E(\omega)$ displays three characteristic peaks above a flat background (Supplementary Fig. 2 c). The first one, at a frequency of about 1 Hz corresponds to cells moving in and out of the submovie boxes. The second one, at about 20-40 Hz corresponds to the rotation rate of the cell body Ω_b, while the third, at about 200 Hz, corresponds to the rotation rate of the flagellum. The location of the maxima of the peaks is estimated via local Gaussian fits and used as a measure of the average rotation speeds within the sample.

Supplementary Note 2

Detailed description of model for flagella propulsion

The model extends on classical force and torque balance analysis of mono-flagellated propulsion [7,8], and accounts for our measurements of swimming speeds as well as cell body and flagellar rotation frequencies. A schematic of the model for flagellar rotation is provided in Supplementary Fig. 5a-c. We assume that the *N* flagella of the cell bundle together tightly, based on experimental evidences for WT cells [9]. Hence, the bundle is described as a helix of increased thickness $r_f = r\,N^{1/2}$. This assumption is based on experiments and simulations showing that the fluid flow generated by two tightly joined helices is equivalent to the fluid flow generated by one helix with a thicker radius [10,11]. We also account for the increased length of the flagella at higher induction of *flhDC*.

Since no net torque or force are applied on the system, the friction forces and torques applying on the flagellar bundle (F_f, Γ_f) and the cell body (F_b, Γ_b) equilibrate in the low Reynolds number fluid:

$$\begin{aligned} F_f + F_b &= 0 \\ \Gamma_f + \Gamma_b &= 0 \end{aligned} \tag{2.1}$$

The friction forces and torques on the flagellar bundle, modelled as a helix, relate to its rotation speed Ω_f and the free-swimming speed U of the cell via

$$\begin{pmatrix} F_f \\ \Gamma_f \end{pmatrix} = \begin{pmatrix} \mu_{TT}^f & -\mu_{TR}^f \\ -\mu_{TR}^f & \mu_{RR}^f \end{pmatrix} \begin{pmatrix} U \\ \Omega_f \end{pmatrix} \tag{2.2}$$

Where μ_X^f are the friction coefficient for translation ($X = TT$), rotation ($X = RR$) and rotation-translation coupling resulting from the flagellum being chiral ($X = TR$). We model the cell body as an (achiral) rod that wobbles. The forces and torques exerted on it relate to body rotation speed Ω_b and swimming speed U via

$$\begin{pmatrix} F_b \\ \Gamma_b \end{pmatrix} = \begin{pmatrix} \mu_{T,b}\,U \\ \mu_{R,b}\,\Omega_b \end{pmatrix} \tag{2.3}$$

Where $\mu_{T,b}$ and $\mu_{R,b}$ are translational and rotational effective friction coefficients. The expressions of the coefficients as a function of the bundle and body geometric parameters are determined below (Eqs. 2.12-2.17 for the flagellum, and 2.19 and 2.38, together with 2.20-2.21, 2.34-2.35 and 2.45, for the cell body).

We assume that the motors all rotate at the same speed Ω_m, which we assume constant based on our observations (i.e., presumably at the maximum rotation speed):

$$\Omega_f - \Omega_b = \Omega_m = \Omega_{Max} \tag{2.4}$$

The set of equations (2.1-2.4) solves readily and provides relations between the swimming speed and the motor rotation speed:

$$U = Z_U\,\Omega_m \tag{2.5}$$

The proportionality coefficient Z_U depends on the number of flagella N and their length and reads:

$$Z_U = \frac{\mu_{TR}^f}{\mu_{T,b} + \mu_{TT}^f} \frac{1}{1 + \mu_{R,\text{red}}^f / \mu_{R,b}} \tag{2.6}$$

With the reduced flagellar rotational friction coefficient

$$\mu_{R,\text{red}}^f = \mu_{RR}^f - \frac{\left(\mu_{TR}^f\right)^2}{\mu_{T,b} + \mu_{TT}^f} \tag{2.7}$$

Additionally, we obtain the following expressions for the swimming speed and the cell body rotation speed relative to the flagellar rotation speed:

$$\frac{U}{\Omega_f} = \frac{\mu_{TR}^f}{\mu_{T,b} + \mu_{TT}^f} \tag{2.8}$$

$$\frac{|\Omega_b|}{\Omega_f} = \frac{\mu_{R,\text{red}}^f}{\mu_{R,b}} \tag{2.9}$$

These ratios are plotted in Supplementary Fig. 5e, f for our experimental measurements compared with the resistive force theory (RFT) predictions, which account for both the length and number of flagella increasing (see below). The qualitative agreement is overall excellent. Consequently, the RFT predicts very well the dependence of swimming speed on flagella number (Supplementary Fig. 5d).

The torque generated by the motors relate to flagellar and cell body torques as:

$$\left|\Gamma_f\right| = |\Gamma_b| = N\Gamma_m \tag{2.10}$$

Which, combined with Eqs. (2.1-2.3), yields:

$$\Gamma_m = \frac{1}{N} \frac{\mu_{R,\text{red}}^f}{1 + \mu_{R,\text{red}}^f / \mu_{R,b}} \Omega_m \tag{2.11}$$

The predicted torque generated by each motor thus decreases as a function of N (Supplementary Fig. 5i), and never exceeds 700 pN.nm, far from the ~1000-2000 pN.nm maximal torque that the motor can generate. Although the model neglects solid friction between filaments, these torque values imply that the motor operates close to or at the maximum speed limit of the torque-speed relation, which is consistent with our modeling hypothesis and our observations of the motor speed being constant, presumably maximal, for all strains.

Expression of friction coefficients

Flagellum

The friction coefficients of the flagellum modeled as a helix are [8,12-14]:

$$\mu_{TT}^f = K_n \, l \sin\psi \, \left(\tan\psi + \frac{\gamma_k}{\tan\psi}\right) \tag{2.12}$$

$$\mu_{TR}^f = K_n \, l \, R \sin\psi \, \left(1 - \gamma_k\right) \tag{2.13}$$

$$\mu_{RR}^{f} = K_n\, l\, R^2 \sin\psi \left(\frac{1}{\tan\psi} + \gamma_k \tan\psi\right) \quad (2.14)$$

With, for Fig. 2e, the coefficients

$$\tan\psi = 2\pi R/\lambda \quad (2.15)$$

$$K_n = 4\pi\eta/\left(\ln\frac{c\lambda}{r_f} + 0.5\right) \quad (2.16)$$

$$\gamma_k = 0.7 \quad (2.17)$$

where the helix parameters are as indicated on Supplementary Fig. 5a, and the medium viscosity is η. The length of the helix l varied between 5 and 8.5 µm, according to measured values in the different strains (Fig. 2), which were least-square fitted as $l = 5.3983 + 3.6564 \log_{10} N$ to quantify the dependence of flagellar length on flagellar number. The helix wavelength $\lambda = 2.3$ µm, radius $R = 0.2$ µm, and flagellar filament thickness $r = 0.01$ µm do not depend on N and are taken from previous measurements [9,13,15]. The bundle thickness is $r_f = r\, N^{1/2}$ to account for the increased cross-section of the bundle.

The coefficient c in Eq. (2.15) takes various values through the literature [8,14], from $c = 2$ in Gray and Hancock (1955)[12] to $c = 0.18/\cos\psi$ in Lighthill (1976)[13], which accounts for some of the hydrodynamic coupling throughout the helix. Similarly, the coefficient $\gamma_k = K_t/K_n$ measures the ratio of the transverse and normal friction coefficients of the rod elements. The coefficient K_t is $K_t = 2\pi\eta/\left(\ln\frac{2\lambda}{r_f} - 0.5\right)$ in Gray and Hancock work[12], and Lighthill[13] recommends to use the expression $K_t = 2\pi\eta/\left(\ln\frac{0.18\lambda}{r_f\cos\psi}\right)$. These give $\gamma_k \simeq 0.5 - 0.6$ in our range of parameters, which strongly overestimates the swimming speed. We indeed tested both full theories and variations where γ_k is assigned a fixed value (Supplementary Fig. 5 and Supplementary Table 1 for the tested models). All variants showed the same qualitative behavior as a function of increasing number of flagella, which matches the experimental trend. It shows that our conclusion that the mechanics of swimming sets a cap on swimming speed at high flagella numbers is solid. However, the full models of both Gray & Hancock and Lighthill were systematically overestimating the speeds, as expected from previous works[8,14]. Nonetheless, we found that using an effective model with Lighthill's formula for K_n, i.e. $c = 0.18/\cos\psi$, and a fixed $\gamma_k = 0.7$ as in (2.17), gave a good quantitative agreement. We thus used this particular model in Fig. 2e of the main text.

Cell body

We model the cell body as a rod of length L, diameter d, aspect ratio $p = L/d$, using typical values for our strain (Supplementary Table 1). The rod is inclined with an angle θ relative to the direction of propulsion ($\mathbf{u}_z$) and we define the angles φ, relative to the x-axis, and α, accounting for the intrinsic cell body rotation about its own axis ($\mathbf{u}_r$) and the associated spherical coordinate system ($\mathbf{u}_r, \mathbf{u}_\theta, \mathbf{u}_\varphi$) (Supplementary Fig. 5b). We also define the associated cylindrical coordinate system $\left(\mathbf{u}_\rho = (\sin\theta\, \mathbf{u}_r + \cos\theta\, \mathbf{u}_\theta), \mathbf{u}_\varphi, \mathbf{u}_z\right)$. We first consider translational friction, before moving to rotations.

<u>Translational friction</u>

The friction force resulting from translation at speed U along the z-axis is given by:

$$F_z = \mathbf{F}\cdot\mathbf{u}_z = \mathbf{u}_z\cdot\left(\zeta_\parallel^T\mathbf{u}_r\mathbf{u}_r + \zeta_\perp^T\mathbf{u}_\theta\mathbf{u}_\theta\right)\cdot(U\,\mathbf{u}_z) = \left[\zeta_\parallel^T + \left(\zeta_\perp^T - \zeta_\parallel^T\right)\sin^2\theta\right]U \tag{2.18}$$

Hence the effective translation friction coefficient of the cell body is:

$$\mu_{T,b} = \zeta_\parallel^T + \left(\zeta_\perp^T - \zeta_\parallel^T\right)\sin^2\theta \tag{2.19}$$

with the translational friction coefficients in the direction parallel and perpendicular to the rod [16]:

$$\zeta_\parallel^T = 2\pi\eta L/(\ln p - 0.207 + 0.980/p - 0.133/p^2 + o(1/p^2)) \tag{2.20}$$

$$\zeta_\perp^T = 4\pi\eta L/(\ln p + 0.839 + 0.185/p + 0.233/p^2 + o(1/p^2)) \tag{2.21}$$

The friction force also has a component perpendicular to the axis of motion in direction $\boldsymbol{u}_\rho$

$$F_\rho = \mathbf{F}\cdot\mathbf{u}_\rho = \mathbf{u}_\rho\cdot\left(\zeta_\parallel^T\mathbf{u}_r\mathbf{u}_r + \zeta_\perp^T\mathbf{u}_\theta\mathbf{u}_\theta\right)\cdot(U\,\mathbf{u}_z) = \left[\left(\zeta_\perp^T - \zeta_\parallel^T\right)\sin\theta\cos\theta\right]U \tag{2.22}$$

Which however averages out over one period of cell body rotation, since $\mathbf{u}_\rho$ rotates. Moreover, in our case, it is negligible compared to the z-component because $\zeta_\perp^T$ is only 20% larger than $\zeta_\parallel^T$, and $\sin\theta\cos\theta \leq 0.1$, as we will see further down.

Rotational friction

To analyze rotational friction properly, we need to account carefully for the balance of torques and the action of the flagella on the cell body. We assume that the N motors connecting the rod to the N flagella are situated on the side of the rod, at positions $\Delta\mathbf{r}_i = l_i\mathbf{u}_r + R\mathbf{u}_i$, with the orientation of the motor:

$$\mathbf{u}_i = \cos(\alpha_i - \alpha_0)\,\mathbf{u}_\theta + \sin(\alpha_i - \alpha_0)\,\mathbf{u}_\varphi \tag{2.23}$$

where α_0 is the intrinsic orientation of the rod and α_i the angular position of the motor on the surface of the cell. By definition, each motor generates a differential of torque between the body and the flagellum, in the direction $\mathbf{u}_i$:

$$\mathbf{\Gamma}_{\mathrm{M}i\to\mathrm{f}i} = -\mathbf{\Gamma}_{\mathrm{M}i\to\mathrm{b}} = \Gamma_M\mathbf{u}_i \tag{2.24}$$

We assume here that all motors deliver the same torque differential Γ_M, and thus that they rotate at the same speed. Since flagella rotate counterclockwise as seen from outside, we have $\Gamma_M > 0$. We assume that torques are balanced on each flagellar filaments individually:

$$\mathbf{\Gamma}_{\mathrm{M}i\to\mathrm{f}i} + \mathbf{\Gamma}_{\mathrm{b}\to(\mathrm{f}i+\mathrm{M}i)} + \mathbf{\Gamma}_{\mathrm{w}\to\mathrm{f}i} = 0 \tag{2.25}$$

Equation (2.25) expresses the fact that at the motor, the body might create a compensation torque $\mathbf{\Gamma}_{\mathrm{b}\to(\mathrm{f}i+\mathrm{M}i)}$ on the flagellum if the motor torque and the friction torque on the flagellum do not compensate because of the curvature of the hook (Supplementary Fig. 5c). By the third Newton's law, a reciprocal torque thus applies on the body at $\boldsymbol{r}_i$:

$$\mathbf{\Gamma}_{(\mathrm{f}i+\mathrm{M}i)\to\mathrm{b}} = \mathbf{\Gamma}_{\mathrm{M}i\to\mathrm{f}i} + \mathbf{\Gamma}_{\mathrm{w}\to\mathrm{f}i} \tag{2.26}$$

The latter adds to the torque that the motor creates on the body. We consider that the flagella are identical and thus:

$$\mathbf{\Gamma}_{\mathrm{w}\to\mathrm{f}i} = \frac{\mathbf{\Gamma}_{\mathrm{w}\to\mathrm{Nf}}}{N} = \frac{\Gamma_{\mathrm{w}\to\mathrm{Nf}}}{N}\mathbf{u}_z \tag{2.27}$$

Moreover, because of the balance of forces on the flagellum, the latter applies at point $\boldsymbol{r}_i$ on the body a force

$$\mathbf{F}_{\mathrm{f}i\to\mathrm{b}} = \mathbf{F}_{\mathrm{w}\to\mathrm{f}i} = \frac{F_{\mathrm{w}\to\mathrm{Nf}}}{N}\mathbf{u}_z \tag{2.28}$$

Here, $\Gamma_{\mathrm{w}\to\mathrm{Nf}}$ and $F_{\mathrm{w}\to\mathrm{Nf}}$ are the rotational and translational friction on the flagellar bundle given by Equation (2.2). Finally, we model the hook as a lossless universal joint [17], which means $\Gamma_M = |\mathbf{\Gamma}_{\mathrm{M}i\to\mathrm{f}i}| = |\mathbf{\Gamma}_{\mathrm{w}\to\mathrm{f}i}| = \Gamma_{\mathrm{w}\to\mathrm{Nf}}/N$.

The balance of torque on the cell body, measured on its center of mass, is therefore:

$$\sum_i \left(\mathbf{\Gamma}_{\mathrm{M}i\to\mathrm{b}} + \mathbf{\Gamma}_{(\mathrm{f}i+\mathrm{M}i)\to\mathrm{b}} + \Delta\mathbf{r}_i \times \mathbf{F}_{\mathrm{f}i\to\mathrm{b}}\right) + \mathbf{\Gamma}_{\mathrm{w}\to\mathrm{b}} = 0 \tag{2.29}$$

Given Equations (2.24), (2.27) and (2.28), Equation (2.29) simplifies into

$$\Gamma_{\mathrm{w}\to\mathrm{Nf}}\,\mathbf{u}_z + F_{\mathrm{w}\to\mathrm{Nf}}\,\langle\Delta\mathbf{r}_i \times \mathbf{u}_z\rangle_i + \mathbf{\Gamma}_{\mathrm{w}\to\mathrm{b}} = 0 \tag{2.30}$$

Note that this expression is valid for any motor distribution $\Delta\mathbf{r}_i$. In our model of flagellar motors along the cell body:

$$\langle\Delta\mathbf{r}_i \times \mathbf{u}_z\rangle_i = -[\langle l_i\rangle_i \sin\theta + R\cos\theta\,\langle\cos(\alpha_i-\alpha_0)\rangle_i]\,\mathbf{u}_\varphi + R\langle\sin(\alpha_i-\alpha_0)\rangle_i\mathbf{u}_\rho \tag{2.31}$$

using the expression for $\Delta\mathbf{r}_i$.

Accounting for the three rotation frequencies $\dot\varphi = \Omega_b$, which is measurable by DFFM, $\dot\alpha$ about the axis $\mathbf{u}_r$, which is not since it does not affect the scattering of light by the cell body, and $\dot\theta$, the pulsation vector is:

$$\boldsymbol{\omega} = \dot\varphi\,\mathbf{u}_z + \dot\alpha\,\mathbf{u}_r + \dot\theta\,\mathbf{u}_\varphi = \dot\varphi\,(-\sin\theta\,\mathbf{u}_\theta + \cos\theta\,\mathbf{u}_r) + \dot\alpha\,\mathbf{u}_r + \dot\theta\,\mathbf{u}_\varphi \tag{2.32}$$

Given the asymmetry in the friction coefficients of a rod, we can write the friction torque that the cell body exerts on the fluid ($\mathbf{\Gamma}_{\mathrm{b}\to\mathrm{w}} = -\mathbf{\Gamma}_{\mathrm{w}\to\mathrm{b}}$) for this rotation as:

$$\mathbf{\Gamma}_{\mathrm{b}\to\mathrm{w}} = \left(\zeta_\parallel^R\mathbf{u}_r\mathbf{u}_r + \zeta_\perp^R\mathbf{u}_\theta\mathbf{u}_\theta + \zeta_\perp^R\mathbf{u}_\varphi\mathbf{u}_\varphi\right)\cdot\boldsymbol{\omega} = \zeta_\parallel^R(\cos\theta\,\dot\varphi + \dot\alpha)\mathbf{u}_r - \zeta_\perp^R\sin\theta\,\dot\varphi\,\mathbf{u}_\theta + \zeta_\perp^R\dot\theta\,\mathbf{u}_\varphi \tag{2.33}$$

The rotational friction coefficients about both axes read [16,18]:

$$\zeta_\perp^R = \frac{\pi}{3}\eta L^3/\left(\ln p - 0.662 + 0.917/p - 0.050/p^2 + o(1/p^2)\right) \tag{2.34}$$

$$\zeta_\parallel^R = \frac{3.841\pi}{4}\eta L d^2\left(1 + 1.119\times10^{-4} + 0.6884/p + 0.2019/p^2 + o(1/p^2)\right) \tag{2.35}$$

Projecting equation (2.30) along the $\mathbf{u}_z$ axis together with equation (2.33), we get:

$$\Gamma_{\mathrm{w}\to\mathrm{Nf}} = \left(\left(\zeta_\parallel^R\cos^2\theta + \zeta_\perp^R\sin^2\theta\right)\dot\varphi + \left(\zeta_\parallel^R\cos\theta\,\dot\alpha\right)\right) \tag{2.36}$$

Searching for a solution that obeys the additional constraints $\dot\alpha = 0$ and $\dot\theta = 0$ yields

$$\Gamma_{\mathrm{w}\to\mathrm{Nf}} = \left(\zeta_\parallel^R\cos^2\theta + \zeta_\perp^R\sin^2\theta\right)\dot\varphi \tag{2.37}$$

This gives the expression of effective rotational friction coefficient, by identification with (2.3)

$$\mu_{R,b} = \zeta_{\parallel}^{R} \cos^2\theta + \zeta_{\perp}^{R} \sin^2\theta = \zeta_{\parallel}^{R} + \left(\zeta_{\perp}^{R} - \zeta_{\parallel}^{R}\right)\sin^2\theta \tag{2.38}$$

Cell orientation

The angle θ is determined by the fact that no net torque is applied perpendicular to the axis $\mathbf{u}_z$. The projections on these directions $\mathbf{u}_\rho$ and $\mathbf{u}_\varphi$ yield respectively:

$$F_{\mathrm{w}\to\mathrm{Nf}}\, R\langle\sin(\alpha_i - \alpha_0)\rangle_i = \sin\theta\cos\theta\left(\zeta_{\parallel}^{R} - \zeta_{\perp}^{R}\right)\dot{\varphi} \tag{2.39}$$

$$\zeta_{\perp}^{R}\dot{\theta} = 0 = -F_{\mathrm{w}\to\mathrm{Nf}}\left[\langle l_i\rangle_i \sin\theta + R\cos\theta\,\langle\cos(\alpha_i - \alpha_0)\rangle_i\right] \tag{2.40}$$

Since the motors are experimentally observed to be evenly spaced along the cell body [19], we make the realistic approximation that $\langle l_i\rangle_i = 0$, hence:

$$\langle\cos(\alpha_i - \alpha_0)\rangle_i = \langle\cos\alpha_i\cos\alpha_0 + \sin\alpha_i\sin\alpha_0\rangle_i = 0 \tag{2.41}$$

This gives the intrinsic cell body orientation:

$$\alpha_0 = -\arctan\left(\frac{\langle\cos\alpha_i\rangle_i}{\langle\sin\alpha_i\rangle_i}\right) \tag{2.42}$$

Equation (2.39) gives:

$$\frac{\sin\theta\cos\theta\left(\zeta_{\perp}^{R} - \zeta_{\parallel}^{R}\right)}{\left(\zeta_{\parallel}^{R} + \left(\zeta_{\perp}^{R} - \zeta_{\parallel}^{R}\right)\sin^2\theta\right)} = -\frac{RF_{\mathrm{w}\to\mathrm{Nf}}}{\Gamma_{\mathrm{w}\to\mathrm{Nf}}}\langle\sin(\alpha_i - \alpha_0)\rangle_i \tag{2.43}$$

This equation is difficult to invert in the general case. However, since $\frac{RF_{\mathrm{w}\to\mathrm{Nf}}}{\Gamma_{\mathrm{w}\to\mathrm{Nf}}} \sim 0.1$ and $\frac{\zeta_{\parallel}^{R}}{\zeta_{\perp}^{R} - \zeta_{\parallel}^{R}} \sim 1$ for our typical flagella and cell body parameters and $\langle\sin(\alpha_i - \alpha_0)\rangle_i < 1$, we can expect $\sin\theta$ to be quite small, which yields at first order:

$$\frac{1}{2}\sin 2\theta \simeq \sin\theta \simeq -\frac{\zeta_{\parallel}^{R}}{\zeta_{\perp}^{R} - \zeta_{\parallel}^{R}}\frac{RF_{\mathrm{w}\to\mathrm{Nf}}}{\Gamma_{\mathrm{w}\to\mathrm{Nf}}}\langle\sin(\alpha_i - \alpha_0)\rangle_i \tag{2.44}$$

Further using equations (2.2) and (2.8) to estimate $F_{\mathrm{w}\to\mathrm{Nf}}/\Gamma_{\mathrm{w}\to\mathrm{Nf}}$, we get at first order:

$$\sin\theta \simeq -\langle\sin(\alpha_i - \alpha_0)\rangle_i \frac{\zeta_{\parallel}^{R}}{\zeta_{\perp}^{R} - \zeta_{\parallel}^{R}} \frac{R\,\mu_{TR}^{f}}{\mu_{RR}^{f} - \dfrac{{\mu_{TR}^{f}}^{2}}{\mu_{TT}^{f} + \tilde{\mu}_{T,b}}} \frac{\tilde{\mu}_{T,b}}{\mu_{TT}^{f} + \tilde{\mu}_{T,b}} \tag{2.45}$$

With $\tilde{\mu}_{T,b} = \frac{\zeta_{\parallel}^{T} + \zeta_{\perp}^{T}}{2}$ as a first approximation of $\mu_{T,b} = \zeta_{\parallel}^{T} + \left(\zeta_{\perp}^{T} - \zeta_{\parallel}^{T}\right)(\sin\theta)^2$ since $\zeta_{\perp}^{T} \simeq \zeta_{\parallel}^{T}$ in our range of parameters.

Model implementation

We computed the model predictions in Matlab. Since the angle θ is determined by the positions of the motors on the cell body, it is expected to vary from cell to cell. For each given number of flagella and model of the flagellum, we thus simulated a population of cells for which these parameters can vary. The friction coefficients of the flagella are first computed from Equations (2.12)-(2.14) with the model specific K_n and γ_k. We then draw for each cell a body length and diameter uniformly within their expected range,

and motor orientations $\{\alpha_i\}$ are drawn from a uniform distribution in [0, 2π]. We get the ζ friction coefficients of the rods from Equations (2.20), (2.21), (2.34) and (2.35). We use Eq. (2.42) and Eq. (2.45) to compute $\sin\theta$, from which we get $\mu_{T,b}$ from Equation (2.19), and $\mu_{R,b}$ from Equation (2.38). We then output the swimming speed and rotation rates from Equations (2.5), (2.8) and (2.9) for each cell. We finally compute for a given number of flagella the population average and standard deviation of U, U/Ω_f, $|\Omega_\mathrm{b}|/\Omega_f$ and $\sin\theta$. The model parameters are summarized in Supplementary Table 1.

Additional discussion

Wobbling angle

Importantly, the angle θ is determined by the positions of the motors on the cell body, and therefore varies from cell to cell. Its population-averaged value, assuming a random distribution, is also a decreasing function of the number of motors (Supplementary Fig. 5g), thus reducing the effective coefficients $\mu_{R,b}$ and $\mu_{T,b}$.

The predicted angle θ is somewhat lower ([1° – 8°]) than what our and previous experimental observations suggest [20,21]. This probably comes from neglecting the elasticity of the hook [17]), which brings additional terms in Eqs. (2.34) and (2.35) that should increase the value of θ but make the analysis significantly more complex. Varying the value of θ by hand within a realistic range of [0° – 30°] leads to only small quantitative and no qualitative change in the behaviour (Supplementary Fig. 5h).

Tight bundle assumption

At high flagella number, the assumption of a tight bundle might start to break down. Previous experiments and simulations on macroscopic helices suggest that the thrust coefficient should not be affected, but translational and, to a lesser extent, rotational friction should increase [11]. This could contribute to the observed moderate decrease of swimming speed (Fig. 1d and Supplementary Fig. 1d) and the saturation of Ω_b/Ω_f (Supplementary Fig. 5f) at high flagella number, which are not fully captured by our model.

Regime of motor operation during swimming

The motor torque we infer from the model is fairly low and suggests that the cells indeed operate in the low-torque / high-speed regime of the flagellar motor torque-speed relationship. This is consistent with the behavior of swimming speed and cell body rotation rates as the viscosity of the suspension increases. These speeds decrease only slightly until a viscosity about three times the viscosity of water [5,22], before turning to a much sharper decrease for higher viscosities, indicating that the motor torque in water should be about 1/3 of the torque at the kink of the relationship (which is 10-20% lower than the maximum torque the motor can deliver [23]).

Our measurements of the cell body and flagellar rotation speeds further imply that the motor speed is constant (220 Hz) as a function of the number of flagella. A first explanation would be that this speed is the maximum speed of the motor. This is consistent with the low torque and in reasonable agreement with previous measurements of this speed (albeit in a slightly different strain), when we interpolate between ω_M(T=22.7 °C) ≈ 300 Hz and ω_M(T=17.7 °C)≈180 Hz [23], for the temperature of our experimental room, which is air-conditioned at T = 20.5±0.5 °C. However, we cannot rule out that solid friction between flagella increases the torque per motor to the level of a single flagellum or that additional mechanisms

regulate the motor speed in swimming cells somewhat below this maximum, e.g. via the dynamics of stator units or such mechanisms as ycgR-based regulation [24].

Supplementary Table 1. Model parameters

	Parameter	Mean value	Range
Body	*L* (µm)	2.5	± 0.4
	d (µm)	0.8	± 0.1
	p	*L* / *d*	
Flagella bundle	λ (µm)	2.3	±0
	R (µm)	0.20	±0
	l (µm)	$5.3983 + 3.6564 \log_{10} N$	±0
	r (nm)	10	±0
	r_f	$r N^{0.5}$	
	ψ	$\arctan 2\pi R/\lambda$	

		Formula	
		K_n	K_t
Model	Lighthill	$4\pi\eta/\left(\ln\frac{0.18\,\lambda}{r_f \cos\psi} + 0.5\right)$	$2\pi\eta/\left(\ln\frac{0.18\lambda}{r_f \cos\psi}\right)$
	Lh $\gamma_k = 0.6$	$4\pi\eta/\left(\ln\frac{0.18\,\lambda}{r_f \cos\psi} + 0.5\right)$	$\gamma_k = 0.6,\ K_t = \gamma_k K_n$
	Lh $\gamma_k = 0.7$	$4\pi\eta/\left(\ln\frac{0.18\,\lambda}{r_f \cos\psi} + 0.5\right)$	$\gamma_k = 0.7,\ K_t = \gamma_k K_n$
	Gray Hancock	$4\pi\eta/\left(\ln\frac{2\lambda}{r_f} + 0.5\right)$	$2\pi\eta/\left(\ln\frac{2\lambda}{r_f} - 0.5\right)$
	GH $\gamma_k = 0.6$	$4\pi\eta/\left(\ln\frac{2\lambda}{r_f} + 0.5\right)$	$\gamma_k = 0.6,\ K_t = \gamma_k K_n$
	GH $\gamma_k = 0.7$	$4\pi\eta/\left(\ln\frac{2\lambda}{r_f} + 0.5\right)$	$\gamma_k = 0.7,\ K_t = \gamma_k K_n$

Supplementary Figures

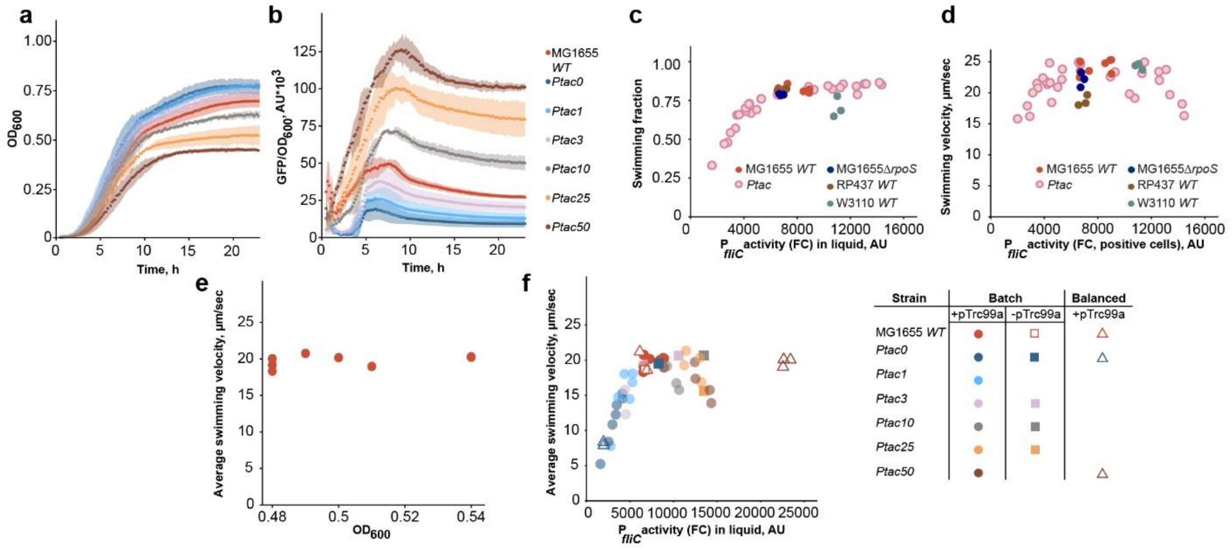

Supplementary Fig. 1 | The effect of flagellar gene expression on growth and motility of *E. coli* K-12 strains in nutrient-rich medium (TB). Cell growth (**a**) and P_{fliC} reporter activity (GFP/OD_{600}) (**b**) were monitored in the indicated cultures of MG1655 *WT* (IL28, see Supplementary Data 1) or its *Ptac* derivative (IL29) for 24 h by measuring absorbance (OD_{600}) and GFP fluorescence every 10 min in the plate reader. Numbers represent the corresponding IPTG concentration for the *Ptac* strain. Standard deviation is shown by the shaded area around the curves ($n = 3$ biological replicates, mean ± s.d.). Changes in the swimming fraction (**c**) and the swimming velocity of motile cells (**d**) as a function of reporter activity (flow cytometry, FC) measured in the indicated *E. coli* K-12 strains (IL28, IL29, IL121, IL149, and IL146). P_{fliC} activity was determined as median GFP intensity in the whole population (**c**) or only in GFP-positive cells (**d**) (see Methods for details). The same range of IPTG concentrations as indicated on panels **a**, **b** was used to induce the expression in *Ptac* strain. Motility and reporter expression were measured separately for each replicate culture (indicated by individual symbols). **e**, Dependence of the population-averaged swimming velocity on OD_{600} for MG1655 *WT* (IL28) grown in TB medium. **f**, Dependence of the population-averaged cell swimming velocity on the activity of the P_{fliC} reporter in the cultures of MG1655 *WT* and *Ptac* strains (with pTrc99a empty vector; IL28 and IL29, respectively) in batch (dots) or under balanced exponential growth (triangles), or in batch without pTrc99a (IL182 and IL183, respectively; squares). The data for batch culture condition (+ pTrc99a) are from Fig. 1d. IPTG concentrations used for *Ptac* strain are depicted by different colors as indicated. Each point represents a single replicate culture. Source data are provided as a Source Data file.

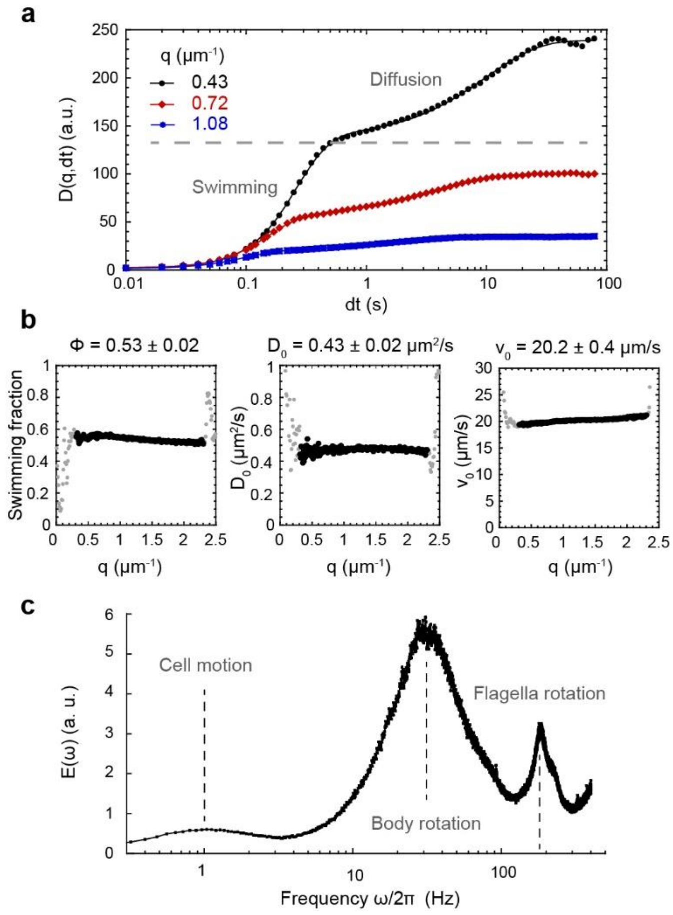

Supplementary Fig. 2 | Differential dynamics microscopy analysis of cell motility. **a, b,** Example measurement for the *Ptac* strain (IL29) at 0 µM IPTG induction. **a**, Differential intensity correlation functions (DICF) as a function of the lag time dt, for different values of the wave number q. The dashed gray line indicates the separation between the contribution of swimming (short times) and diffusion (long times) to the increase of the DICF for q=0.43 µm^{-1}. Points are experimental data and lines are fits by the (swimming + diffusion of non-swimmers) model (see Supplementary Note 1). **b**, Resulting fit parameters (fraction of swimmers ϕ, diffusion coefficient D_0, and average velocity v_0) as a function of q. Dark dots indicate successful fits and gray dots are the ones that fail due to either lack of full decorrelation (small q) or low signal over noise (large q). Consistent fit parameter values over the valid range of q validate the model. The mean and standard deviation of the fit parameter values over the valid range are indicated. **c**, Example of normalized power spectrum $E(\omega)$ obtained by dark field flicker microscopy (DFFM) for MG1655 *WT* (IL28) cells. The second and third peaks measure the rotation frequencies of the cell body and flagellum, respectively, while the first comes from cell motion across the measurement box. Source data are provided as a Source Data file.

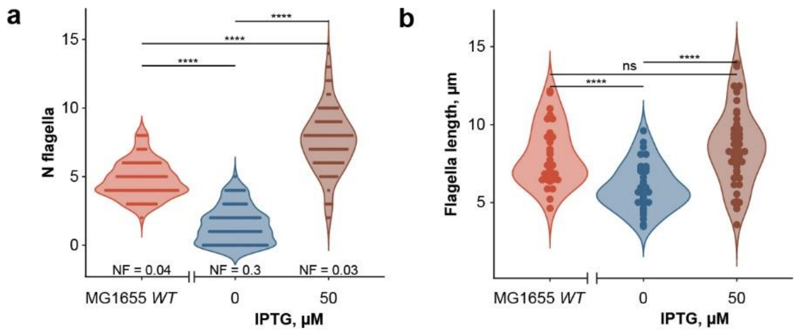

Supplementary Fig. 3 | Distributions of flagellar number and length in the population of MG1655 *WT* (IL28) and *Ptac* (IL29) strains (0, 50 μM IPTG). Each point on the violin plot is a single-cell measurement of flagellar number (**a**, *n* = 106 cells from multiple fields of view) or length (**b**, *n* = 35, 46, 47 flagellar filaments in 10, 20 and 7 cells of MG1655 *WT* (IL28), *Ptac0* and *Ptac50* (IL29), respectively for the indicated condition. The analysis of flagellar lengths was done using different numbers of cells to ensure that the number of filaments is comparable between conditions. Normality of means was tested by the Shapiro-Wilk test ($P \leq 0.05$ (**a**), and $P \geq 0.05$ (**b**)). Due to the large sample size ($n > 20$), a two-sided t-test was used for both (**a**) and (**b**) to compare the differences between the population means. Since the hypothesis of equal variances was rejected (Levene's test, $P \leq 0.05$), we used Welch's t-test followed by the Holm-Bonferroni method to correct for multiple testing, and the adjusted *P* values (****$P \leq 0.0001$) are shown on both panels. NF on (**a**) indicates the fraction of non-flagellated cells for each condition. Source data are provided as a Source Data file.

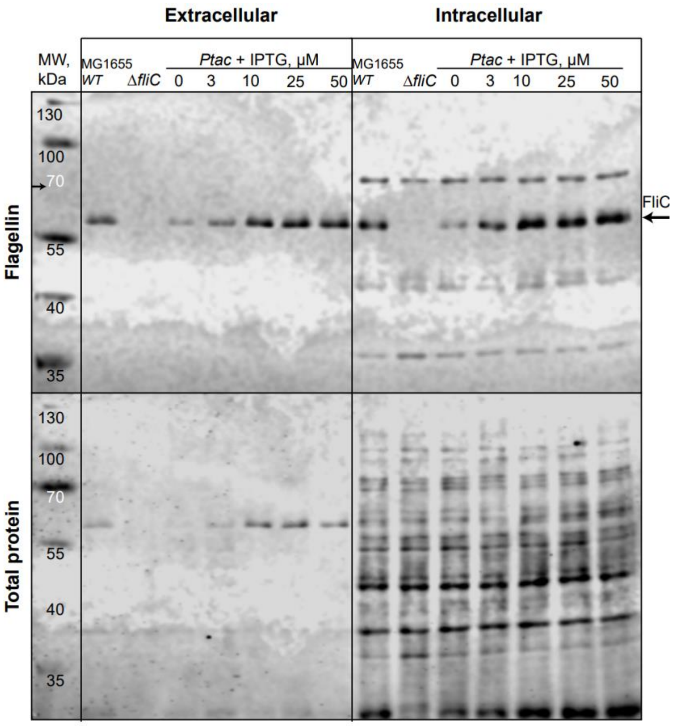

Supplementary Fig. 4 | The amount of intra- and extracellular flagellin increases as a function of flagellar gene expression. Immunoblotting analysis of flagellin (FliC, indicated by black arrow) in intra- and extracellular fractions of MG1655 *WT* (IL28), *Ptac* (IL29) and *ΔfliC* (VS575, negative control) cells. Sample volumes were adjusted by OD_{600} normalization prior to loading. Membrane staining for total protein was used as a loading control (bottom). MW, kDa – band profile of the prestained protein ladder; the black arrow indicates the 70 kDa band which is not visible in 800 nm channel used for flagellin detection. See Methods for details.

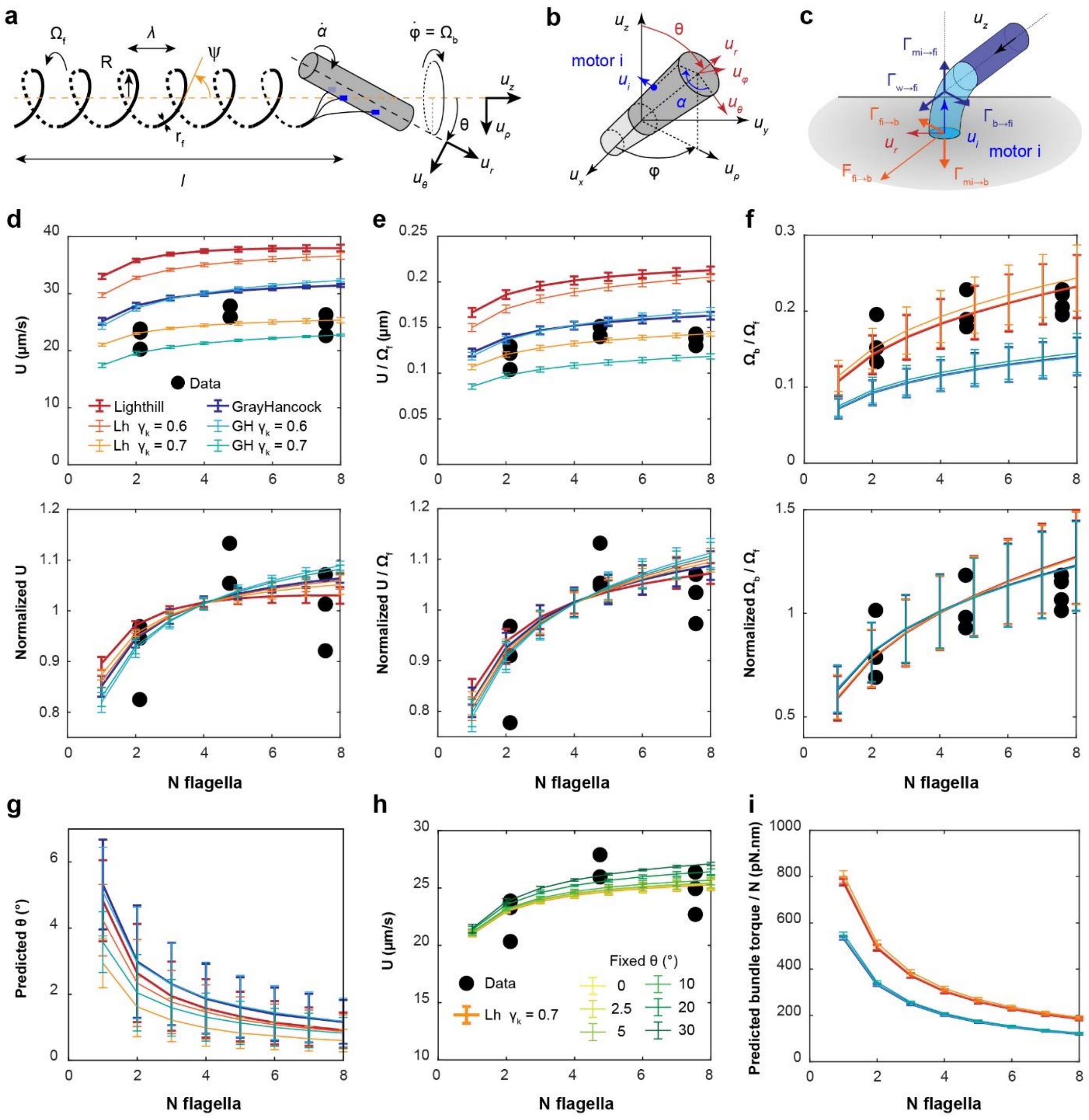

Supplementary Fig. 5 | Model of flagellar propulsion. **a**, Schematic illustration of the model of flagellar propulsion, with the tightly wrapped flagellar bundle rotated by N motors and counter-rotating elongated cell body. Geometric parameters (defined in Supplementary Note 2) as well as body (Ω_b) and flagellar (Ω_f) rotation speeds are indicated. The swimming direction is ***u*z**. **b**, Schematic of the cell body with the system of reference for the implantation of the motors. **c**, Balance of forces (F) and torques (Γ) applying on the motor and flagellum at the motor i. **d-f**, Predicted swimming speed U (**d**), U/Ω_f (**e**) and Ω_b/Ω_f (**f**) by the force balance analysis (Supplementary Note 2, Eqs. 2.5-2.6 (**d**), 2.8 (**e**), and 2.9 (**f**)) for the indicated variants of resistive force theory, which predicts flagella friction coefficients

(Supplementary Note 2), compared to experimental data for *Ptac0* (IL29), MG1655 *WT* (IL28) and *Ptac50* (IL29) strains (from left to right; each black dot represents an individual biological replicate) using DDM and DFFM (see Supplementary Note 1). Top: Absolute values, Bottom: Values normalized by the mean of the data or the prediction over the range *N* flagella = [1, 8]. In (**d**), the motor rotation speed is set to 220 Hz. The model with the best quantitative match (Lh, γ_k=0.7) is shown in Fig. 2e. **g**, Verifications of model assumptions, with predicted tilt angle θ between the cell body and the direction of motion, which is smaller than typical experimental values (10-30°)[25,26], likely due to the neglected hook elasticity. **h,** Predictions for the model presented in Fig. 2e (Lh, γ_k=0.7), where the tilt angle θ is set to the indicated values instead of being predicted by the model, showing that any relatively small angle is compatible with the experimental data. **i**, Predicted torque on flagellar bundle divided by the number of motors N for all models (Supplementary Note 2, Eq. 2.11). (**d-i**) The error bars represent the standard deviation over n=5000 modeled cells with cell body size and motor orientations drawn randomly from expected distributions (see Supplementary Note 2 for details). (**d-g, i**) Colors represent the models as indicated in panel d, which are fully defined in Supplementary Table 1 and Supplementary Note 2. Source data are provided as a Source Data file.

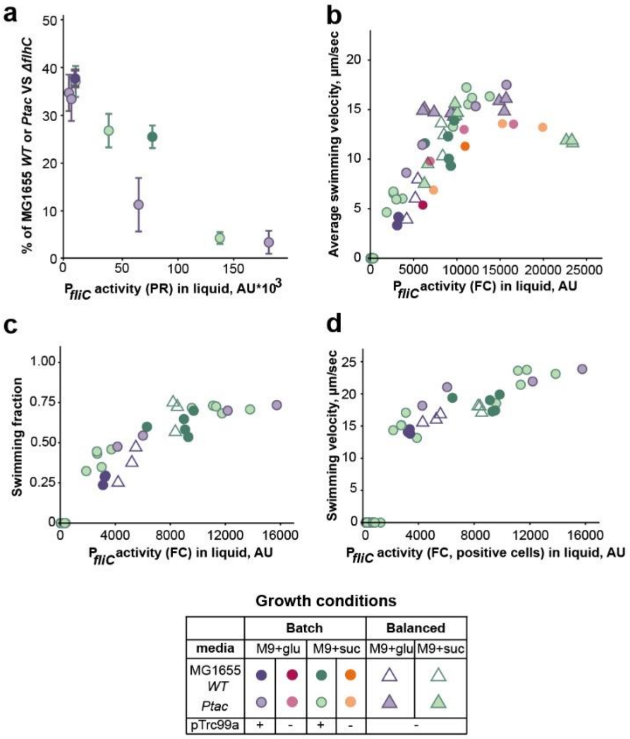

Supplementary Fig. 6 | The impact of flagellar gene expression on growth (a) and motility (b-d) in minimal medium. a, Growth fitness cost of motility in M9 glucose (M9+glu) and M9 succinate (M9+suc). Strains were initially co-inoculated in a 1:1 ratio, and fitness cost was determined as the percentage of either MG1655 *WT* (IL26) or *Ptac* (IL107) strain (induced with 1-25 µM IPTG in M9+glu or 1-10 µM IPTG in M9+suc) (labeled with CFP) in the co-cultures with the non-flagellated *ΔflhC* strain (labeled with YFP, IL25) after 72 h of incubation with shaking (200 rpm). P_{fliC} activity measured in the plate reader (PR) was used to plot the data. The mean ± s.d. values (*n* = 3 biological replicates) are shown. **b**, Dependence of the population-averaged swimming velocity on flagellar gene expression in the cultures of MG1655 *WT* and *Ptac* strains with (IL28, IL29) and without (IL182, IL183) an empty pTrc99a vector grown in batch (dots) or under balanced exponential growth (triangles), as indicated in the table below. The range of IPTG concentrations used for *Ptac* strain was the same as in panel **a**. Each point is an independent replicate culture. Changes in the swimming fraction (**c**) and the swimming velocity of motile cells (**d**) as a function of reporter activity (flow cytometry, FC) measured in the indicated strains grown in M9+glu or M9+suc. P_{fliC} activity was determined as median GFP intensity in the whole population (**c**) or only in GFP-positive cells (**d**) (see Methods for details). Source data are provided as a Source Data file.

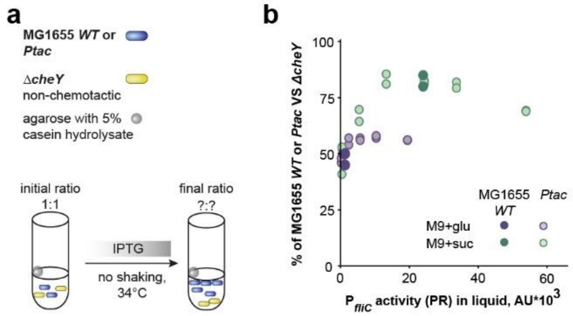

Supplementary Fig. 7 | Growth fitness benefit of flagellar gene expression in minimal medium. Schematic overview (**a**) and results (**b**) of pairwise growth competition between chemotactic MG1655 *WT* (IL26) or *Ptac* (IL107) strain (induced by different concentrations of IPTG) (labeled with CFP) and non-chemotactic *ΔcheY* strain (labeled with YFP, IL27) grown in the presence of localized nutrient source (agarose beads containing 12 % of casein hydrolysate) for 72 h without shaking. Strains were initially co-inoculated in a 1:1 ratio, and fitness benefit was quantified as the percentage of MG1655 *WT* or *Ptac* strain in the mixed population at the end of the experiment (*n* = 2 biological replicates). P_{fliC} activity measured in the plate reader (PR) was used to plot the data (*n* = 1). Note that a different plate reader was used in these experiments, for consistency with a previous study by Ni et al.[27] (see Methods). Source data are provided as a Source Data file.

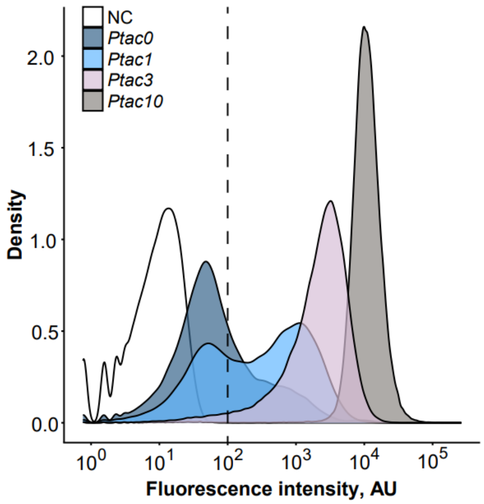

Supplementary Fig. 8 | Flow cytometry measurements of P_{flic}-GFP reporter activity in the *Ptac* (IL29) cell population grown in M9 succinate. Flagellar gene expression was induced by different concentrations of IPTG (indicated by numbers); the *Ptac* strain lacking the reporter plasmid (VS1683) served as negative control (NC). The vertical dashed line indicates the threshold P_{flic} activity level for cellular auto-fluorescence defined as the signal from cells without any fluorescent reporters used to distinguish GFP-positive from GFP-negative cells**.** Source data are provided as a Source Data file.

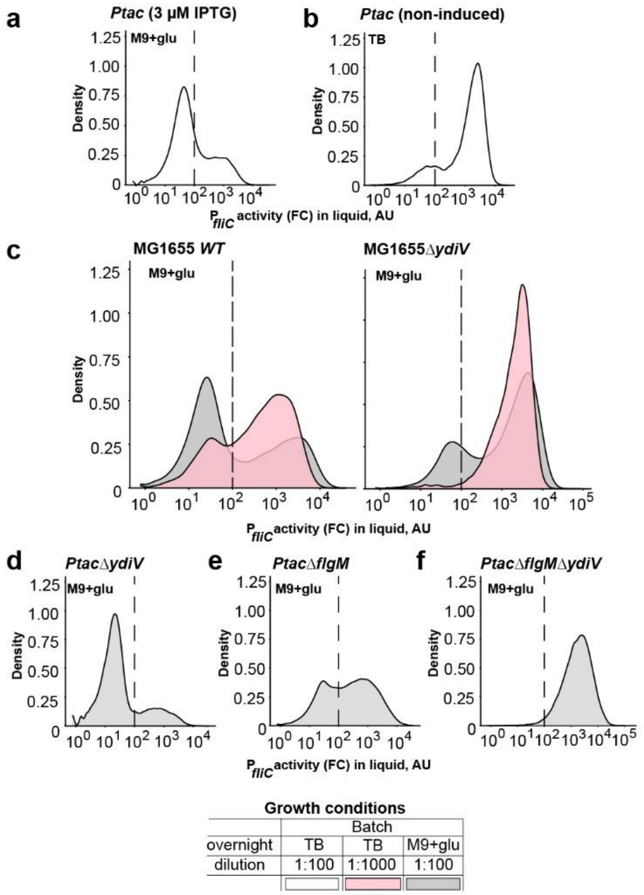

Supplementary Fig. 9 | Flow cytometry analysis of P_{fliC}-GFP reporter activity in the populations of the indicated *E. coli* strains under different growth conditions. As stated in the legend table (bottom), cell cultures were prepared by diluting (1:100 or 1:1000) a TB- or M9 glucose-grown (M9+glu) overnight in the target medium, as indicated. Distribution of P_{fliC}-GFP activity levels in the population of *Ptac* (IL29) strain induced by 3 µM of IPTG and grown in M9+glu (**a**), a non-induced *Ptac* (IL29) strain grown in TB (**b**). Distribution of P_{fliC}-GFP activity levels in the populations of MG1655 *WT* (IL28) and MG1655*ΔydiV* (IL164) (**c**), non-induced *PtacΔydiV* (IL165) (**d**), *PtacΔflgM* (IL175) (**e**) and *PtacΔflgMΔydiV* (IL217) (**f**) strains grown in M9 glucose. The vertical dashed line indicates threshold P_{fliC} activity level for cellular auto-fluorescence defined as the signal from cells without any fluorescent reporters. Representative data from one biological replicate are shown. Source data are provided as a Source Data file.

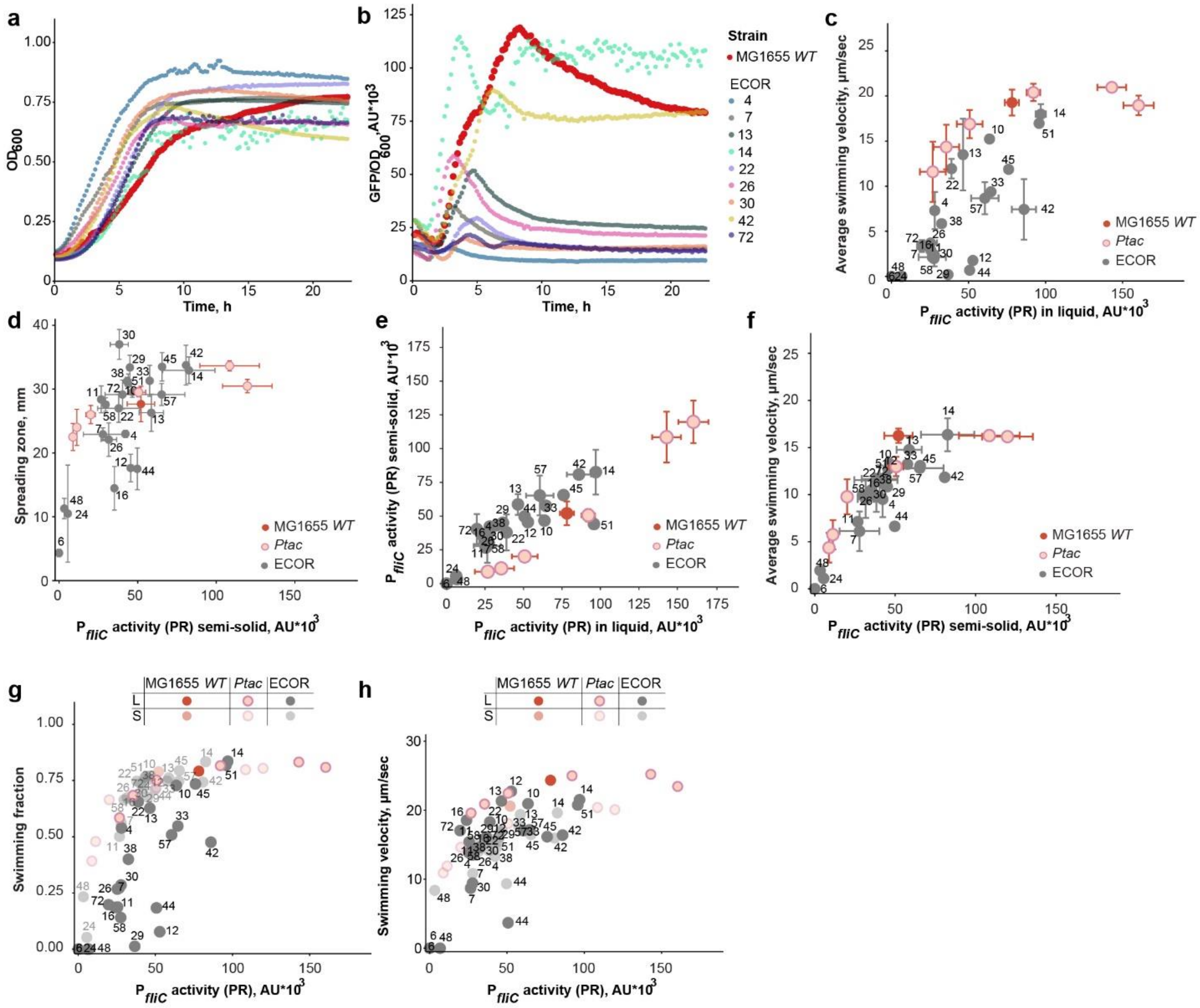

Supplementary Fig. 10 | Growth, P_{fliC} reporter activity, and motility of MG1655 *WT*, *Ptac* and the cohort of 24 natural *E. coli* isolates. Cell growth (**a**) and P_{fliC} reporter activity (GFP/OD_{600}) (**b**) for MG1655 *WT* (IL28) and the indicated ECOR strains grown in TB medium. Starting cultures were prepared by dilution (1:100) of a TB-grown overnight. Cells were grown at 30°C for 24h and both absorbance (OD_{600}) and GFP fluorescence were measured every 10 min in the plate reader. Representative data for one biological replicate are shown. **c**, Dependence of the population-averaged swimming velocity on P_{fliC} reporter activity in liquid TB medium. **d**, Dependence of the average spreading zone diameter (in mm) in porous 0.27% TB agar on P_{fliC} reporter activity in *E. coli* strains grown on semi-solid 0.5% TB agar. **e**, Correlation between P_{fliC} reporter activity in liquid and on the semi-solid TB medium. **f**, Dependence of the population-averaged swimming velocity on the reporter activity on the semi-solid TB medium. On c-f, shown are the data from Fig. 4 for 10 ECOR strains, MG1655 *WT* (IL28) and *Ptac* (IL29) strains measured in replicates (n = 3, mean ± s.d.) , together with the non-replicate measurements for the remaining 14 ECOR strains. Dependence of the fraction of motile cells (**g**) and the swimming velocity of these cells (**h**) on P_{fliC} reporter activity of the indicated *E. coli* strains grown in liquid TB medium (L, bright dots) or on semi-solid TB agar (S, pale dots). Shown

are the mean values for the subset of 10 ECOR strains, MG1655 *WT* (IL28) and *Ptac* (IL29) strains measured in replicates together with the non-replicate measurements for the remaining 14 ECOR strains. Source data are provided as a Source Data file.

Supplementary references

1. Cerbino, R. & Trappe, V. Differential dynamic microscopy: probing wave vector dependent dynamics with a microscope. *Phys. Rev. Lett.* **100**, 188102 (2008).
2. Wilson, L.G. *et al.* Differential Dynamic Microscopy of Bacterial Motility. *Phys. Rev. Lett.* **106**, 018101 (2011).
3. Martinez, V.A. *et al.* Differential Dynamic Microscopy: A High-Throughput Method for Characterizing the Motility of Microorganisms. *Biophys. J.* **103**, 1637-1647 (2012).
4. Cerbino, R. & Cicuta, P. Perspective: Differential dynamic microscopy extracts multi-scale activity in complex fluids and biological systems. *J. Chem. Phys.* **147**, 110901 (2017).
5. Martinez, V.A. *et al.* Flagellated bacterial motility in polymer solutions. *Proc Natl Acad Sci U S A.* **111**, 17771-17776 (2014).
6. Cerbino, R., Piotti, D., Buscaglia, M. & Giavazzi, F. Dark field differential dynamic microscopy enables accurate characterization of the roto-translational dynamics of bacteria and colloidal clusters. *J. Phys. Condens. Matter*. **30**, 025901 (2018).
7. Purcell, E.M. The efficiency of propulsion by a rotating flagellum. *Proc Natl Acad Sci U S A.* **94**, 11307-11311 (1997).
8. Chattopadhyay, S., Moldovan, R., Yeung, C. & Wu, X.L. Swimming efficiency of bacterium *Escherichia coli*. *Proc Natl Acad Sci U S A* **103**, 13712-7 (2006).
9. Turner, L., Ryu, W.S. & Berg, H.C. Real-time imaging of fluorescent flagellar filaments. *J. Bacteriol.* **182**, 2793-801 (2000).
10. Kim, M.J. *et al.* Particle image velocimetry experiments on a macro-scale model for bacterial flagellar bundling. *Exp. Fluids* **37**, 782-788 (2004).
11. Danis, U. *et al.* Thrust and Hydrodynamic Efficiency of the Bundled Flagella. *Micromachines* **10**, 449 (2019).
12. Gray, J. & Hancock, G.J. The Propulsion of Sea-Urchin Spermatozoa. *J. Exp. Biol.* **32**, 802-814 (1955).
13. Lighthill, J. Flagellar Hydrodynamics. *SIAM Review* **18**, 161-230 (1976).
14. Johnson, R.E. & Brokaw, C.J. Flagellar Hydrodynamics - Comparison between Resistive-Force Theory and Slender-Body Theory. *Biophys. J.* **25**, 113-127 (1979).
15. Morgan, D.G., Owen, C., Melanson, L.A. & Derosier, D.J. Structure of Bacterial Flagellar Filaments at 11 Angstrom Resolution - Packing of the Alpha-Helices. *J. Mol. Biol.* **249**, 88-110 (1995).
16. Tirado, M.M., Martinez, C.L. & Delatorre, J.G. Comparison of Theories for the Translational and Rotational Diffusion-Coefficients of Rod-Like Macromolecules - Application to Short DNA Fragments. *J. Chem. Phys.* **81**, 2047-2052 (1984).
17. Bianchi, S., Saglimbeni, F., Frangipane, G., Cannarsa, M.C. & Di Leonardo, R. Light-Driven Flagella Elucidate the Role of Hook and Cell Body Kinematics in Bundle Formation. *PRX Life* **1**, 013016 (2023).
18. Tirado, M.M. & Garcia de la Torre, J. Rotational-Dynamics of Rigid, Symmetric Top Macromolecules - Application to Circular-Cylinders. *J. Chem. Phys.* **73**, 1986-1993 (1980).
19. Li, H. & Sourjik, V. Assembly and stability of flagellar motor in *Escherichia coli*. *Mol. Microbiol.* **80**, 886-899 (2011).

20. Darnton, N.C., Turner, L., Rojevsky, S. & Berg, H.C. On torque and tumbling in swimming Escherichia coli. *J. Bacteriol*. **189**, 1756-1764 (2007).
21. Patteson, A.E., Gopinath, A., Goulian, M. & Arratia, P.E. Running and tumbling with *E. coli* in polymeric solutions. *Sci*. *Rep.* **5**(2015).
22. Qu, Z.J. & Breuer, K.S. Effects of shear-thinning viscosity and viscoelastic stresses on flagellated bacteria motility. *Phys*. *Rev*. *Fluids*. **5**, 073103 (2020).
23. Chen, X. & Berg, H.C. Torque-speed relationship of the flagellar rotary motor of *Escherichia coli*. *Biophys. J*. **78**, 1036-41 (2000).
24. Boehm, A. *et al.* Second Messenger-Mediated Adjustment of Bacterial Swimming Velocity. *Cell* **141**, 107-116 (2010).
25. Darnton, N.C., Turner, L., Rojevsky, S. & Berg, H.C. On torque and tumbling in swimming Escherichia coli. *J. Bacteriol*. **189**, 1756-64 (2007).
26. Patteson, A.E., Gopinath, A., Goulian, M. & Arratia, P.E. Running and tumbling with E. coli in polymeric solutions. *Sci. Rep.* **5**, 15761 (2015).
27. Ni, B., Colin, R., Link, H., Endres, R.G. & Sourjik, V. Growth-rate dependent resource investment in bacterial motile behavior quantitatively follows potential benefit of chemotaxis. *Proc Natl Acad Sci U S A* **117**, 595-601 (2020).
