# Supplementary material for: Physics of swimming and its fitness cost determine strategies of bacterial investment in flagellar motility
Source: Nat Commun. 2025 Feb 18;16:1731. doi: 10.1038/s41467-025-56980-x (PMC11836070; doi:10.1038/s41467-025-56980-x)
Supplement: Supplementary file 4 — Description of Additional Supplementary Files [file 41467_2025_56980_MOESM4_ESM.pdf]

Description of Additional Supplementary Files:

Supplementary Data 1: *Escherichia coli* K-12 strains and plasmids used in the study.

Supplementary Data 2: Motility of natural *Escherichia coli* isolates from ECOR collection.
