# Supplementary material for: Physics of swimming and its fitness cost determine strategies of bacterial investment in flagellar motility
Source: Nat Commun. 2025 Feb 18;16:1731. doi: 10.1038/s41467-025-56980-x (PMC11836070; doi:10.1038/s41467-025-56980-x)
Supplement: Supplementary file 5 — Reporting Summary [file 41467_2025_56980_MOESM5_ESM.pdf]

nature portfolio

Corresponding author(s): Victor Sourjik

Last updated by author(s): Jan 29, 2025

Reporting Summary

Nature Portfolio wishes to improve the reproducibility of the work that we publish. This form provides structure for consistency and transparency in reporting. For further information on Nature Portfolio policies, see our Editorial Policies and the Editorial Policy Checklist.

Statistics

For all statistical analyses, confirm that the following items are present in the figure legend, table legend, main text, or Methods section.

n/a	Confirmed	
☐	☒	The exact sample size (*n*) for each experimental group/condition, given as a discrete number and unit of measurement
☐	☒	A statement on whether measurements were taken from distinct samples or whether the same sample was measured repeatedly
☐	☒	The statistical test(s) used AND whether they are one- or two-sided<br>*Only common tests should be described solely by name; describe more complex techniques in the Methods section.*
☒	☐	A description of all covariates tested
☐	☒	A description of any assumptions or corrections, such as tests of normality and adjustment for multiple comparisons
☐	☒	A full description of the statistical parameters including central tendency (e.g. means) or other basic estimates (e.g. regression coefficient) AND variation (e.g. standard deviation) or associated estimates of uncertainty (e.g. confidence intervals)
☐	☒	For null hypothesis testing, the test statistic (e.g. *F*, *t*, *r*) with confidence intervals, effect sizes, degrees of freedom and *P* value noted<br>*Give P values as exact values whenever suitable.*
☒	☐	For Bayesian analysis, information on the choice of priors and Markov chain Monte Carlo settings
☒	☐	For hierarchical and complex designs, identification of the appropriate level for tests and full reporting of outcomes
☒	☐	Estimates of effect sizes (e.g. Cohen's *d*, Pearson's *r*), indicating how they were calculated

Our web collection on statistics for biologists contains articles on many of the points above.

Software and code

Policy information about availability of computer code

Data collection	ImageJ (v1.49), BD FACSDivaTM Software (v8.0.1), Microsoft Excel, Kaleidagraph (v4.1), ZEN v3.0 (black edition), Matlab R2020a
Data analysis	Data on swimming velocity and flagella rotation were analyzed with custom-written plugins of ImageJ. Additional information is provided in the Supplementary Note 1. The Image analysis plugins have been published previously and are available at https://gitlab.gwdg.de/remy.colin/FourierImageAnalysis<br>For flow cytometry data analysis, the population of interest was first selected during measurements using BD FACSDivaTM Software v8.0.1 (see Methods). The events for only this population were exported as raw FCS files and processed with the flowCore package in R v4.2.2. Summary statistics was collected using "Batch analysis" function of BD FACSDivaTM Software v8.0.1 and processed with basic R functions as well.<br>Swimming parameters (swimming fraction and swimming velocity of swimmers) were extracted from the output txt files generated by ImageJ, which were further processed in R v4.2.2 using custom-written functions. Flagellar rotation data were processed as indicated in Supplementary note 1 in Kaleidagraph (v4.1)<br>To count flagella number, images were first processed in ZEN v3.0 (blue edition). Flagellar lengths were measured in ImageJ using "segmented line" tool. Both measurements were manually transferred into Excel files and summary statistics was using rstatix package in R v4.2.2 (details are provided in the respective figure legend).<br>Most plots were generated with a custom-written R script (R version 4.2.2) using packages from the tydiverse library.

For manuscripts utilizing custom algorithms or software that are central to the research but not yet described in published literature, software must be made available to editors and reviewers. We strongly encourage code deposition in a community repository (e.g. GitHub). See the Nature Portfolio guidelines for submitting code & software for further information.

Data

Policy information about availability of data

All manuscripts must include a data availability statement. This statement should provide the following information, where applicable:

- Accession codes, unique identifiers, or web links for publicly available datasets
- A description of any restrictions on data availability
- For clinical datasets or third party data, please ensure that the statement adheres to our policy

All data are available in the main text or in Supplementary Information. Source Data are provided with the manuscript. All materials are available from the corresponding author upon request.
Matlab codes for the model are available at https://gitlab.gwdg.de/remy.colin/RFTMultiflagella. The plugins for DDM (previously published) and DFFM are available at https://gitlab.gwdg.de/remy.colin/FourierImageAnalysis

Research involving human participants, their data, or biological material

Policy information about studies with human participants or human data. See also policy information about sex, gender (identity/presentation), and sexual orientation and race, ethnicity and racism.

Reporting on sex and gender	no human participants, their data, or biological material was used
Reporting on race, ethnicity, or other socially relevant groupings	no human participants, their data, or biological material was used
Population characteristics	no human participants, their data, or biological material was used
Recruitment	no human participants, their data, or biological material was used
Ethics oversight	no human participants, their data, or biological material was used

Note that full information on the approval of the study protocol must also be provided in the manuscript.

Field-specific reporting

Please select the one below that is the best fit for your research. If you are not sure, read the appropriate sections before making your selection.

☒ Life sciences ☐ Behavioural & social sciences ☐ Ecological, evolutionary & environmental sciences

For a reference copy of the document with all sections, see nature.com/documents/nr-reporting-summary-flat.pdf

Life sciences study design

All studies must disclose on these points even when the disclosure is negative.

Sample size	In most cases, sample sizes were determined based on previous studies. To quantify flagella number, 106 cells per condition were analyzed, including all the cells from each field of view. The analysis of flagellar lengths was done using different numbers of cells to ensure that the number of filaments is comparable between conditions. All the cells from each field of view were included in the measurements
Data exclusions	No data were excluded
Replication	Unless specified otherwise, all experiments were performed at least three times. Multiple microscopy images of one biological replicate were used for the measurements of flagellar lengths and numbers
Randomization	Random bacterial colonies were picked for measuring spreading zones in soft TB agar
Blinding	The only part of our study where blinding could have been applied is the measurement of flagellar lengths and numbers. However, since we analyzed all the cells from each field of view, blinding was not relevant

Reporting for specific materials, systems and methods

We require information from authors about some types of materials, experimental systems and methods used in many studies. Here, indicate whether each material, system or method listed is relevant to your study. If you are not sure if a list item applies to your research, read the appropriate section before selecting a response.

Materials & experimental systems

n/a	Involved in the study
☐	☒ Antibodies
☒	☐ Eukaryotic cell lines
☒	☐ Palaeontology and archaeology
☒	☐ Animals and other organisms
☒	☐ Clinical data
☒	☐ Dual use research of concern
☒	☐ Plants

Methods

n/a	Involved in the study
☒	☐ ChIP-seq
☐	☒ Flow cytometry
☒	☐ MRI-based neuroimaging

Antibodies

Antibodies used: anti Flagellin rabbit polyclonal FITC conjugate primary antibody (Antikoerper; AA 2-498-FITC, ABIN2831532, 1.5 mg/ml), IRDye 800CW Donkey Anti-Rabbit IgG secondary antibody (LI-COR Biosciences; P/N: 926-32213, 1 mg/ml). Both antibodies were used at 1:10000 dilution

Validation: For the primary antibody AA 2-498-FITC, validation was done by the manufacturer (https://www.antikoerper-online.de/antibody/1992301/anti-Flagellin+FliC+AA+2-498+antibody+FITC/). Negative control was included in the analysis (Supplementary Fig. 4)

Plants

Seed stocks: no plant material was used

Novel plant genotypes: no plant material was used

Authentication: no plant material was used

Flow Cytometry

Plots

Confirm that:

☐ The axis labels state the marker and fluorochrome used (e.g. CD4-FITC).

☒ The axis scales are clearly visible. Include numbers along axes only for bottom left plot of group (a 'group' is an analysis of identical markers).

☐ All plots are contour plots with outliers or pseudocolor plots.

☒ A numerical value for number of cells or percentage (with statistics) is provided.

Methodology

Sample preparation: Mono- and co-cultures of bacteria expressing different fluorescent markers were prepared as described in Methods.

Instrument: BD LSRFortessa SORP cell analyzer (BD Biosciences, Germany)

Software: BD FACSDivaTM Software v8.0.1, flowCore package in R v4.2.2

Cell population abundance: The samples were prepared by diluting the highly abundant cell cultures (see Methods). 30,000 cells (events) were recorded and analyzed in each experimental run

Gating strategy: All gating strategies we used are described in the Methods. Gating was first performed on an FSC-A/SSC-A plot and on an SSC-W over SSC-H plot to exclude doublets. Events in the samples with fluorescence intensities higher than the background signal from the MG1655 WT or Ptac strain without the reporter plasmid were considered 'positive' (illustrated in Supplementary Fig. 8)

☒ Tick this box to confirm that a figure exemplifying the gating strategy is provided in the Supplementary Information.
